# Do Changes in the Pace of Events Affect One-Off Judgments of Duration?

**DOI:** 10.1371/journal.pone.0059847

**Published:** 2013-03-28

**Authors:** Hannah M. Darlow, Alexandra S. Dylman, Ana I. Gheorghiu, William J. Matthews

**Affiliations:** Department of Psychology, University of Essex, Colchester, United Kingdom; University of Melbourne, Australia

## Abstract

Five experiments examined whether changes in the pace of external events influence people’s judgments of duration. In Experiments 1a–1c, participants heard pieces of music whose tempo accelerated, decelerated, or remained constant. In Experiment 2, participants completed a visuo-motor task in which the rate of stimulus presentation accelerated, decelerated, or remained constant. In Experiment 3, participants completed a reading task in which facts appeared on-screen at accelerating, decelerating, or constant rates. In all experiments, the physical duration of the to-be-judged interval was the same across conditions. We found no significant effects of temporal structure on duration judgments in any of the experiments, either when participants knew that a time estimate would be required (prospective judgments) or when they did not (retrospective judgments). These results provide a starting point for the investigation of how temporal structure affects one-off judgments of duration like those typically made in natural settings.

## Introduction

The rate or speed of external events influences people’s estimates of duration. In particular, intervals that are filled with more events or faster-moving objects are usually judged longer than those with fewer events or slower speeds [1]–[5]. However, relatively few studies have asked how *changes* in the pace of external events influence our judgment of time. In an early study, Buffardi [6] found that clustering events near the beginning of an interval led to longer subjective duration than clustering them near the end (see also [7], [8]). More recently, Matthews [9] found that shapes moving with constant speed were judged longer than those which decelerate, which in turn seemed longer than those which accelerate. Similarly, a recent series of studies using brief tone sequences found that evenly-spaced sequences were judged longer than those with a change in tempo [10]. Moreover, temporal structure interacted with physical duration: accelerating sequences were judged longer than decelerating ones at short durations but the pattern reversed at longer durations.

These studies suggest that changes in tempo affect judgments of duration, but all were conducted in the psychophysical tradition: the stimuli were meaningless, lasted at most a few seconds, and participants judged many items over the course of the experiment. There is increasing emphasis on duration estimates produced under more ecologically valid circumstances (e.g., [11]–[13]). The current work examines the effects of temporal structure on duration estimates under more natural conditions, where participants made one-off judgments of intervals lasting a few minutes. That is, each participant judged a single time interval defined by a meaningful event sequence. Understanding such judgments is practically important because they more closely resemble the time estimates made in everyday life (people rarely judge the durations of hundreds of tones one after the other but are often asked how long a single experience or task lasted). Moreover, one-off judgments of long intervals may well be made different from psychophysical judgments because (a) judgment of intervals longer than about 1 second are often argued to rely on cognitive and neural mechanisms which are different from those subserving judgment of shorter intervals (see [14] for a review), and (b) psychophysical tasks elicit comparisons between stimuli presented in the experimental session, which can exert profound context effects on temporal estimates [10], [15]–[17], whereas one-off judgments minimize such effects.

For some participants in our experiments, duration estimates were *retrospective* (participants did not know that they would be asked for a duration judgment). For others, the judgments were *prospective* (participants were forewarned that they would be asked for a duration judgment). Previous research suggests that these two types of judgment draw upon different mechanisms and are differentially affected by experimental manipulations (e.g., [18]). Experiments 1a–1c had participants judge pieces of music whose physical duration was always the same but whose tempo either gradually increased (accelerating condition), gradually decreased (decelerating condition), or remained steady (constant-rate condition). Experiment 2 used a visuo-motor task in which participants responded to left and right arrows which appeared at increasing, constant, or decreasing rates, with total duration and number of stimuli held constant. Experiment 3 used a reading task in which participants read “fun facts” which appeared at increasing, decreasing, or constant rates, with the total duration and number of items equated across conditions. Competing theories of temporal judgment make different predictions about the effects of temporal structure in these studies.

One class of theory emphasises memory processes and is particularly relevant to retrospective judgments. In particular, it has been argued that remembered duration is positively related to the “storage size” [19], degree of segmentation [20], [21] or number of contextual changes [22] during the interval. Within these frameworks, accelerating, decelerating, and constant-rate stimuli might be expected to have the same remembered duration because they comprise the same number of events. On the other hand, changes in tempo are presumably an integral part of the overall “stimulus complexity” and may necessitate changes in processing style over the course of the interval, in which case accelerating and decelerating items will be judged longer than constant-rate stimuli. An alternative memory-based perspective comes from Boltz [23], [24], who has suggested that highly coherent stimuli (where the temporal and non-temporal structure affords high predictability) are more successfully encoded into memory, yielding more accurate duration estimates. One plausible idea is that gradual changes in tempo will reduce coherence by making the hierarchical structure of the event sequence less apparent, thereby reducing accuracy for the accelerating and decelerating items.

A second class of theory concerns "active timing". When people know that a temporal judgment will be required (in a prospective judgment task), they can employ intentional timing strategies during stimulus presentation. Many theorists assume that intentional timing is based on some kind of counting or accumulation process, often involving the flow of pulses from a pacemaker [25]–[30]. If the pacemaker keeps a steady rate then temporal judgment will be unaffected by temporal structure (assuming that other aspects of the process – such as the latency to begin/end the accumulation of pulses – are also unaffected). However, several authors have argued that the pacemaker is not constant but is affected by factors such as stimulus modality and intensity (e.g., [31]–[33]). In particular, presenting a stream of clicks at 5 Hz for a few seconds seems to speed up the pacemaker, lengthening the subjective duration of a subsequent stimulus [32]–[34] (see also [35]). This implies that the pacemaker rate is coupled to the tempo of external stimulation. If so, the nature of this coupling will determine the effect of temporal structure on judged duration. A linear coupling predicts no effect because the average tempo is the same for accelerating, decelerating, and constant rate conditions. However, some authors have suggested a negatively-accelerated (e.g., logarithmic) relationship between pacemaker rate and the pace of stimulus change [9], [10], [36]. Under this view, judged duration will be maximal for stimuli with constant rate [4]. Beckman and Young [1] have suggested an alternative, change-based model in which stimulus change combines additively with physical duration (a proxy for degree of environmental change) to determine subjective time. A simple view equates change with number of events or distance travelled, in which case accelerating, decelerating, and constant-rate stimuli which are matched on these dimensions will appear to have equal duration. If second-order change is also taken into account, then accelerating and decelerating sequences will be judged longer than constant-tempo ones. Other authors have emphasized the role of attention in active timing (e.g., [37]–[39]), although the predicted effects of changes in tempo under these accounts are unclear. Finally, although memory-based and active-timing accounts are most obviously applicable to retrospective and prospective judgment tasks respectively, it is worth noting that participants might nonetheless use memory-based strategies for prospective judgments and rely on incidental pacemaker-based timing for retrospective judgment.

The five experiments described here examine the effects of temporal structure on one-off judgments of duration using both prospective and retrospective judgment tasks, and aim to constrain the theoretical accounts outlined above.

## Experiment 1a

Experiment 1a had participants listen to a piece of music whose tempo either accelerated, decelerated, or remained constant, and then make an unexpected judgment of the music’s duration.

### Method

All Experiments were approved by the Faculty Ethics Committee for the University of Essex Faculty of Science and Engineering. Experiments 2 and 3 obtained written consent. For Experiments 1a–1c, consent was oral, in keeping with the British Psychological Society Code of Human Research Ethics and approved by the University of Essex Faculty of Science and Engineering ethics committee. Oral consent was appropriate because participation took place in diverse public settings, was anonymous, and comprised a task (listening to a brief piece of piano music and judging how long it lasted) that posed no conceivable risk to the participants, who were free to withdraw at any time. No participants withdrew after starting the task, and participants’ written responses to the study questions was used to document their consent.

#### Participants

An opportunity sample of 135 participants (65 females) aged 19–68 years (*M* = 32.5 years, *SD* = 11.7 years) were recruited from a variety of locations (e.g., on campus, in their workplace).

#### Stimuli

The stimuli were three pieces of piano music: Inventio 4 by Johann Sebastian Bach (in 3/8); an excerpt from Sarabande by Georg Friedrich Händel (in 3/2); and an excerpt from Sonata Facile by Wolfgang Amadeus Mozart (in 4/4). The pieces were chosen to be unfamiliar to the majority of the participants and were edited in Musescore (www.musescore.org), a freely-available piece of music-composition software. Musecore has an integrated sequencer and synthesizer which plays musical scores and allows the resulting performances to be saved as midi files. Pause and repeat signs were removed, and tempos of 78, 96, and 112 beats per minutes were chosen for the Bach, Händel, and Mozart pieces, respectively. Using a synthesizer to generate the pieces has the advantage of avoiding slight changes in tempo/pauses etc that come during human performance.

We used Audacity’s “Silence finder” (audacity.sourceforge.net) to apply a uniform criterion for removing silence from the end of the recordings (specifically, silence was defined as intensity less than −50 dB lasting more than 0.1 seconds). We then used Audacity’s “Sliding time scale/pitch shift” tool to adjust the tempo without altering the pitch. To create the “accelerating” stimuli, we set the starting tempo at 20% below the original value and the final tempo at 20% above the original value. For the “decelerating” stimuli, the starting and final tempos were set at 20% above and below the original value, respectively. These values were selected so that the changes in tempo were gradual but the initial and terminal tempos were very noticeably different.

The accelerating and decelerating versions of each piece had identical durations, but differed by a few hundred milliseconds from the original, constant-tempo pieces (an artefact of the tempo adjustment procedure). We therefore used the sliding time scale tool to apply a very small constant shift to the constant-pace stimuli so that they had the same durations as the corresponding accelerating and decelerating pieces. An advantage of this approach is that all three versions of each piece of music (accelerating, decelerating, constant-tempo) had been subject to the sliding time scale algorithm.

These manipulations resulted in constant-tempo, accelerating, and decelerating versions of all three pieces (Bach, Händel, Mozart). The constant-tempo, accelerating, and decelerating versions of each piece had the same duration, and the pieces were extremely similar in length: (Bach = 61.07 seconds; Händel = 61.05 seconds; Mozart = 61.03 seconds). Finally, we used the Scale Intensity function of Praat (www.fon.hum.uva.nl/praat/) to norm the average nominal intensities to 65 dB. (Even before norming, the nominal intensities were very similar for all pieces: 60.3–61.7 dB). All of the final stimuli were saved as stereo wav files and are available from the authors.

#### Design and procedure

Participants were recruited in a variety of settings. They read an information sheet telling them that the experiment involved listening to a piece of music and that it should take no more than 5 minutes of their time. They were asked to listen carefully to the piece of music over headphones and told that, once it finished, they should remove the headphones, whereupon they would be given a question sheet. Participants were not told in advance that a duration judgment would be required. The music was played from a portable music player over Sennheiser HD 580 headphones. The testing environments typically involved low-level background noise.

Each of the 9 composer×condition combinations was played to 15 different participants. The first question on the response sheet asked: “How long do you think that the piece of music lasted? Go with your intuitive judgment - do not try to use a watch or clock.” There followed space to enter a judgment in minutes and seconds. The next question asked participants to put a small mark on a horizontal line “to indicate how long the piece of music felt to you”, in order to probe their subjective impressions (which can be affected by event structure in a way not always detectable with absolute duration estimates [40]). The line was 14 cm long and labelled “Very short” and “Very long” at the ends. (For the first 20 participants, a formatting error meant that the labelling was slightly ambiguous. However, the experimenter was available to provide verbal clarification, and exclusion of these participants made no difference to the results.) The distance of the participant’s mark from the “Very short” end of the line was measured and divided by the total line length to get a response scaled between 0 and 1.

Participants were also asked to rate their enjoyment of the piece from 1 to 7 (where 1 = not at all and 7 = very much), to indicate whether they recognized the music and, if they did, to try to name the piece. Finally, they indicated their age and gender.

### Results and Discussion

Alpha was set to.05 for all analyses.

#### Recognition

Preliminary analysis showed that 25 participants (18.5%) purported to recognize the piece they were played, distributed fairly evenly over the decelerating (*N* = 12), accelerating (*N = *8) and constant-tempo (*N = *5) conditions, 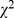
(2, *N* = 135) = 3.63, *p* = .16, and between the pieces by Bach (*N = *9), Händel (*N = *4), and Mozart (*N = *12), 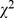
(2, *N* = 135) = 4.81, *p* = .090. Of the people who said they recognized the music, 7 attempted to identify it, mostly by venturing a composer; no-one named the piece correctly but 5 either correctly named the composer or named another piece by the right composer. Thus, it seems that the pieces met the aim of being largely unrecognized, and recognition was unrelated to either composer or temporal structure.

#### Temporal judgments

Here and below, preliminary ANOVAs were conducted which included composer and recognition as between-subject variables. With one exception (noted below), none of the main effects or interactions involving these factors was significant and they were dropped from the analysis. (Their inclusion made no difference to the effects of temporal structure).

Responses to the duration estimation question were converted into seconds and are shown in Table 1. The judgments from the decelerating, accelerating, and constant-tempo conditions did not significantly differ, *F*(2,132) = .71, *p* = .492, 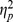 = .01. The duration estimates were significantly greater than the true duration for all three conditions (all *p*s <.001). Table 1 also shows the subjective impression judgments which, like the duration estimates, were unaffected by temporal structure, *F*(2,132) = 2.09, *p* = .128, 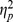 = .03. Levene’s test indicated that the variability of responses did not differ between conditions for either the duration estimates or the subjective impression judgments, *F*(2, 132) = 0.10, *p* = .907 and *F*(2, 132) = 0.27, *p* = .766, respectively.

**Table 1:** Mean responses for Experiments 1a and 1b.

		Duration Estimate			Subjective Impression		
		Dec	Con	Acc	Dec	Con	Acc
Expt 1a	*M*	122.56	118.07	112.60	0.300	0.339	0.361
	*SD*	40.31	39.80	38.69	0.146	0.140	0.146
Expt 1b	*M*	89.42	93.33	80.18	0.390	0.358	0.333
	*SD*	42.05	50.44	38.68	0.164	0.157	0.142

Duration estimates are in seconds. Dec = Decelerating; Con = Constant-tempo; Acc = Accelerating.

#### Enjoyment

A supplementary analysis examined the effects of condition on enjoyment. There was no effect, *F*(2,132) = .44, *p* = .646, 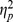 = .01. (Caution may be needed here because there was some heterogeneity of variance, *F*(2,132) = 3.21, *p* = .044).

#### Correlations between measures

We calculated the correlations between enjoyment ratings, duration estimates and subjective judgements to assess the size of any relationship between these variables. The results showed a weak, significant relationship between duration estimate and subjective judgement, *r = *.20, *p* = .023. The other correlations were very small and not significant which suggests extremely weak relationships between duration estimates and enjoyment, *r* = −.11, *p* = .214, and between subjective judgement and enjoyment, *r* = −.005, *p* = .955.

In short, this experiment found no effect of temporal structure on retrospective judgments of music duration.

## Experiment 1b

Experiment 1b was very similar to Experiment 1a, except that participants knew in advance that they would be asked how long the music lasted. In addition, the participant sample was rather different.

### Method

#### Participants

One hundred and thirty five participants (66 female) aged 17–40 years (*M* = 21.3 years, *SD* = 3.0 years) took part. One additional participant reported crackling/loss of signal from the headphones during testing and was replaced. (It is possible that other participants experienced similar problems, but none mentioned it.) Participants were primarily students at the University of Essex, recruited during the term and tested at convenient locations around campus such as in the university library and lobbies (cf Experiment 1a, which used more participants from outside the university and was conducted over the summer vacation). The mean age of the participants is about 10 years younger than in the previous experiment.

#### Stimuli, design, and procedure

The experiment was very similar to Experiment 1a, except that the initial instruction sheet informed participants that they would be asked to judge how long the music lasted, and told them not to use a watch because “we are interested in your intuitive judgment of time”. The response sheet added a question asking participants “how you went about estimating how long the piece of music lasted”. A similar question was asked at the end of Experiments 1c and 2, below. These judgment-strategy data are not analyzed here, but are available from the authors.

### Results and Discussion

#### Recognition

Twenty participants (14.8%) reported recognizing the music, distributed fairly evenly across decelerating (*N* = 10), constant-tempo (*N* = 4), and accelerating (*N* = 6) conditions, 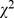
(2, *N = *135) = 3.29, *p* = .193, and between the pieces by Bach (*N* = 7), Händel (*N* = 5), and Mozart (*N* = 8), 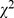
(2, *N* = 135) = 0.82, *p* = .663. Only five participants made any attempt to name the music; of these, one named the correct composer. As before, the pieces were largely unrecognized, as intended.

#### Temporal judgments

The duration estimates for the decelerating, constant-tempo, and accelerating conditions are shown in Table 1, and did not differ, *F*(2,132) = 1.06, *p* = .349, 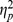 = .02. The estimates were significantly longer than the true duration in all conditions (all *p*s <.001). Table 1 also shows the subjective impression judgments, which were likewise unaffected by condition, *F*(2,132) = 1.52, *p* = .223, 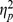 = .02. Levene’s test indicated that the response variability did not differ between conditions for either the duration estimates or the subjective impression judgments, *F*(2, 132) = 0.56, *p* = .571 and *F*(2, 132) = 1.79, *p* = .171, respectively.

#### Enjoyment

The preliminary three-way ANOVA with condition, composer, and recognition as between-subjects factors indicated that the only significant factor affecting enjoyment was recognition: participants who reported recognizing the piece also reported greater enjoyment (*M* = 5.25, *SD* = 1.12) than those who did not (*M* = 4.01, *SD* = 1.43), *F*(2,118) = 13.19, *p*<.001, 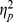 = .10. No other main effects or interactions were significant (all *p*s >.14).

#### Correlations between measures

Duration estimates were only weakly correlated with subjective impressions, and the relationship missed significance: *r* = .167, *p* = .053. Neither type of temporal judgment correlated with enjoyment (for duration estimates: *r* = .04, *p* = .640; for subjective impressions: *r* = −.081, *p* = .349).

In short, Experiment 1b found no effect of temporal structure on one-off prospective judgments of music duration.

## Experiment 1c

Neither the retrospective judgments of Experiment 1a nor the prospective judgments of Experiment 1b were affected by changes in tempo. However, inspection of the mean responses in Table 1 suggests two potentially interesting trends. Firstly, duration estimates were much longer (and therefore less accurate) in the retrospective task (overall mean = 117.7 seconds) than in the prospective task (overall mean = 87.6 seconds). Secondly, in the retrospective task the subjective-impression responses were greatest for the accelerating stimuli and smallest for the decelerating stimuli, whereas in the prospective task the order is reversed.

The results of Experiments 1a and 1b are not directly comparable because participants were not randomly assigned to tasks, and the participant groups were quite different in some respects (e.g., age). Experiment 1c therefore combined prospective and retrospective tasks in a single experiment, to test the reliability of the cross-experiment differences suggested by Table 1. Because the previous experiments indicated that the accelerating and decelerating conditions are most different, we dropped the constant-tempo condition to boost the power of the study.

### Method

#### Participants

Two hundred and four participants aged 18–51 (*M* = 21.7, *SD* = 4.6) took part; 67 were male. Five additional participants were discarded because they indicated that they had previously taken part in a music duration-judgment experiment. Participants were members of the University of Essex participant pool and were paid £3. Most booked test sessions through an on-line system; some were recruited by the experimenter in person.

#### Stimuli, design, and procedure

As before, this experiment used a fully between-subjects design. Participants were tested in a quiet testing cubicle. The accelerating and decelerating versions of the music from each composer were used equally often, half for retrospective judgments and half for prospective judgments. The experimenter cycled through the 12 cells of the design in sequence, with the exception of the last 5 participants who were tested as replacements for earlier participants. Instructions for the retrospective task were as in Experiment 1a; those for the prospective task were the same as for Experiment 1b. The response sheets were similar to before, except that the “enjoyment” question was dropped. At the end, all participants were asked to write how they formed their judgment.

### Results and Discussion

#### Recognition

A total of 26 participants (12.7%) indicated that they recognized the music; they were distributed fairly evenly over accelerating (*N* = 16) and decelerating (*N = *10) conditions, 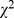
(1, *N* = 204) = 1.59, *p* = .208, and between retrospective (*N* = 11) and prospective (*N* = 15) tasks, 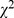 (1, *N* = 204) = .71, *p* = .40; there was some variation in the recognition of pieces by Bach (*N* = 3), Händel (*N* = 8), and Mozart (*N* = 15), 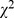
(2, *N* = 204) = 9.61, *p* = .008]. Twelve people wrote something in the box asking them to name the piece; three named the correct composer and one additional person named the correct piece. As in the previous experiments, it seems that the choice of stimuli achieved the goal of being largely unrecognized.

#### Temporal estimates

The duration estimates are shown in Table 2. A 2×2 ANOVA indicated no main effect of condition, *F*(1,200) = 0.03, *p* = .866, 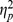 = .00 and no task×condition interaction, *F*(1,200) = 0.57, *p* = .450, 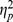 = .00. Although retrospective judgments tended to be longer than prospective judgments, the effect missed significance, *F*(1,200) = 3.51, *p* = .063, 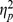 = .02. Mean estimates were significantly above the true duration for all conditions (all *p*s <.001). Subjective impressions were similarly unaffected by condition, task, and their interaction (all *Fs* <1, *p*s >.3). Levene’s test showed that neither duration estimates nor subjective impressions showed differences in variability across the cells of the design, *F*(3, 200) = 1.70, *p* = .169 and *F*(3,200) = 1.15, *p* = .331, respectively. As in the earlier experiments, magnitude estimates and subjective judgments were weakly correlated, *r* = .21, *p* = .003.

**Table 2:** Mean responses for Experiment 1c.

		Duration Estimate		Subjective Impression	
		Dec	Acc	Dec	Acc
Retrospective	*M*	110.25	116.16	.376	.414
	*SD*	39.15	50.99	.169	.148
Prospective	*M*	103.14	99.39	.394	.402
	*SD*	46.36	44.93	.163	.180

Duration estimates are in seconds. Dec = Decelerating; Acc = Accelerating.

Experiment 1c therefore found no evidence for an overall effect of changes in tempo on judgments of music duration and no modulation by judgment task. Experiments 1a–1c used music to define the to-be-judged interval. In addition to its emotive qualities and familiarity, music typically has a highly coherent, hierarchical structure [38], and may be judged different from other types of stimuli/task [41]. The next two experiments generalized the preceding findings to two other types of task, one of which involved active responding (Experiment 2) and one which simply involved processing text (Experiment 3).

## Experiment 2

Experiment 2 examined the effects of changes in the pace of events on the judged duration of a visuo-motor task.

### Methods

#### Participants

A planned sample of 162 participants was tested (115 female, ages 18–55 years, *M* = 21.8 years, *SD* = 5.1 years); two additional participants encountered technical difficulties and had to be replaced. The participants were recruited from the University of Essex participant pool, and were paid £3 each.

#### Design and Procedure

The experiment employed a 2×3 design with judgment task (prospective; retrospective) and condition (constant rate; accelerating; decelerating) manipulated between subjects. Due to a minor coding error, the numbers of participants in the six cells of the design were not equal, but the design was balanced for each factor separately. That is, half of the participants were in the prospective task condition, half were in the retrospective task condition, and one third of the participants were assigned to each temporal structure (constant rate, accelerating, and decelerating).

Participants signed a consent form providing relevant information, including the maximum duration of the study (10 minutes). The main task was computer-based and was performed in a sound-attenuating chamber. Instructions were presented on-screen, telling the participants that they should respond as quickly as possible to arrows that appear on the screen. At this stage, participants in the prospective condition were informed: “At the end, we will ask you how long you spent doing the task”; participants in the retrospective condition were only told “At the end, we will ask you some questions about the task”.

Participants were told that they should press the “S” key if the arrow pointed left and the “K” key if it pointed right, responding as quickly as possible while maintaining accuracy. It was explained that the arrows would keep appearing independently of the participant’s responses, and that they should focus on the screen throughout.

After the last page of instructions, the message “Get ready” was displayed centrally on-screen for 2 seconds, followed by a 2 second blank before the first stimulus. The task consisted of 91 black arrows presented sequentially on a white background, pointing either left or right with the direction randomly chosen on each trial. Stimulus timing was measured in frames (screen refreshes); the monitor had a refresh rate of 85 Hz (therefore one frame was one 85th of a second) and a resolution of 1024×780 pixels. Stimulus presentation and response collection were controlled using DMDX [42].

The arrows were displayed for 200 ms (15 frames) each, followed by a blank screen. The duration of the blank screen between the arrows depended on the condition. In the “constant rate” condition, there was a 2153 ms (183 frames) blank between stimuli. In the “accelerating” condition, the blank was 3212 ms (273 frames) on the first trial and decreased by 23.5 ms (2 frames) with each subsequent trial, reaching 1094 ms (93 frames) on the final trial. In the “decelerating” condition, the blanks followed the reverse pattern of the “accelerating” condition.

Following the final post-stimulus blank, the instructions “End of Task. Wait a moment” were displayed for 1 second (3 participants saw the “End of Task” message for slightly less than the usual 1 second because they pressed a response key when the message was on the screen). The end-of-task message was followed by instructions to estimate task duration (defined as the time from the “Get Ready” message at the start until the “End of Task” message at the end), in minutes and seconds. Participants were told to make intuitive judgements without referring to a watch or clock. A second question requested participants to mention the strategy used to come up with the time estimate. Both responses were made on a paper response sheet.

### Results and Discussion

The actual times between the appearance of the “Get Ready” signal and the appearance of the “End of Task” signal were measured by the computers’ internal clocks. Across all participants, the mean was 215.98 seconds (*SD* = 0.01 seconds); there was minor variation because of occasional dropped frames etc, but across all participants the shortest and longest durations differed by only 66 ms. Accuracy on the visuo-motor task was generally high (*M* = 94.1% correct, *SD* = 9.3%). One participant scored well below chance (2.1% correct), suggesting that they got the response keys the wrong way round.

The data of primary interest were the duration estimates. Each participant gave an estimate of the task duration, in minutes and seconds, which was converted into seconds for analysis purposes. In the few cases when participants reported approximations such as “about 2–3 minutes”, the average of the two values was considered in the analysis, resulting in one score per participant. The mean judgments are shown in Table 3.

**Table 3:** Mean responses for Experiment 2.

		Dec	Con	Acc
Retrospective	*M*	252.25	219.69	231.67
	*SD*	95.86	64.78	98.07
	*N*	28	32	21
Prospective	*M*	247.50	252.73	232.48
	*SD*	93.29	105.53	74.12
	*N*	26	22	33

Duration estimates are in seconds. Dec = Decelerating; Con = Constant-rate; Acc = Accelerating.

A 2×3 fully between-subjects ANOVA was performed to see if the duration estimates depended on task and/or temporal structure. The results showed no main effect of task, *F*(1,156) = 0.48, *p* = .488, 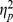 = .00, no main effect of condition, *F*(2,156) = 0.60, *p* = .551, 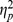 = .01, and no interaction, *F*(2,156) = 0.71, *p = *.492, 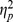 = .01. Levene’s test indicated that the variance of the time estimates was not significantly different across groups, *F*(5,156) = 0.97, *p* = .435. Mean duration judgments were overestimates for all 6 task×condition combinations (all *p*s >.05, but across all participants the overestimation was reliable, *t*(161) = 3.28, *p* = .001).

Since the design of the study was not fully balanced (the cell sizes were only equal for each of the factors taken separately), two additional one-way between-subjects ANOVAs were performed, one for each independent variable. There was no difference in judgements between the accelerating, decelerating, and constant-rate conditions, *F*(2,159) = 0.71, *p* = .494, 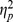 = .01. The non-significant effect of task was also replicated, *F*(1,160) = 0.41, *p* = .524, 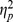 = .00.

In short, there was no difference between the three temporal structure conditions. Moreover, time estimates were not significantly different between participants who were informed they would have to make a duration judgement and those who were not, and the effects of temporal structure were not dependent on whether the participants knew they will be required to make such a judgement.

## Experiment 3

Experiment 3 examined the effects of temporal structure on the judged duration of a reading task, and focused on retrospective judgments.

### Method

#### Participants

A total of 134 native speakers of English (86 females) participated in this experiment. Their mean age was 21.5 years (*SD* = 5.7 years), ranging from 18 to 55 years.

#### Design and Procedure

The stimuli consisted of 16 “facts” taken from www.snopes.com (e.g., “The youngest mother on record was a five-year-old Peruvian girl”), presented in black font on a white background on a CRT monitor (1280×1024 pixels refreshing at 85 Hz). The stimulus presentation was controlled by PsychoPy [43].Participants were tested individually in sound-attenuating cubicles, and were randomly assigned to one of three conditions (constant, accelerating, or decelerating rate). Participants were told that they would be shown a list of “fun facts”, one at a time, that they should read each one while it was on the screen, and that they would be asked some questions about what they had read at the end. They were not informed that a duration estimate would be required.

In the constant-rate condition, the facts were presented for 5 seconds each (425 frames); in the decelerating condition, the first fact was presented for 2 seconds (170 frames), and each subsequent fact was presented for 400 ms (34 frames) longer than the previous, up to 8 seconds (680 frames) for the last one; the accelerating condition was the mirror image of the accelerating condition (e.g., the first fact was presented for 8 seconds, and each subsequent fact was shown for 400 ms shorter than the previous one). In all conditions, the total duration was the same (*M* = 81.6 seconds, *SD* = 0.03, ranging from 81.49 to 81.63 seconds). (Note that the actual time varied very slightly between participants because the duration of one display frame is not always precisely 1/85^th^ of a second.).

The 16 facts were presented in the same order for all participants. Following the presentation of the facts, the participants were asked to judge (without using a watch) the time duration (in seconds) between the appearance of the first fact and the disappearance of the last one. They were further told that the answer lies somewhere between 0 and 180 seconds.

### Results and Discussion

The response from one participant who reported having misunderstood the task was excluded. The mean time judgment was 83.00 seconds (*SD* = 40.18) in the constant-rate condition (*N* = 43), 84.98 seconds (*SD* = 39.46) in the accelerating condition (*N* = 45), and 81.97 seconds (*SD* = 44.19) in the decelerating condition (*N = *45). A one-way between subjects ANOVA revealed no reliable difference in time judgement between the three conditions, *F*(2,130) = 0.06, *p* = .940, 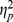 .00, and Levene’s test indicated no heterogeneity of response variance, *F*(2,130) = 0.10, *p* = .901. Mean duration estimates did not differ from the true event duration (for all three conditions, and collapsing over condition, *p*s >.5).

A secondary analysis was conducted excluding 12 participants who reported unusually low judgments of less than 20 seconds (suggesting that they may have misunderstood the instructions). Excluding these from the analysis did not change the effect of condition, *F* <1.

In short, this experiment again found no effect of changes in the pace of events on retrospective judgments of time.

### Conclusions

Across five studies, we found no significant effect of changes in the pace of events on one-off judgments of duration. Table 4 summarizes the effect sizes for the duration estimates in the current experiments (with the values calculated separately for the prospective and retrospective conditions of Experiments 1c and 2). The table shows both eta-squared and omega-squared values, with the latter being regarded as a less biased estimate of the population value [44]. Across all studies, the effect of temporal structure is small. Table 4 also shows the effect sizes for the contrasts between the decelerating, accelerating and (where applicable) constant-rate conditions of each experiment. These estimates were submitted to a random-effects meta-analysis using the metafor package for the R statistical language [45], [46]. None of the contrasts showed significant heterogeneity in effect size (all *p*s >.60) and the overall effect size estimates were −0.104 (95% confidence interval, CI: −0.307,0.100) for the accelerating-constant rate contrast; 0.089 (95% CI: −0.074, 0.253) for the decelerating-accelerating contrast, and 0.057 (95% CI: −0.146, 0.259) for the decelerating-constant rate contrast. Note that all of the confidence intervals are relatively narrow and span zero.

**Table 4:** Effect sizes for all studies.

		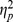	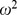	Acc - Con	Dec - Acc	Dec - Con
Expt 1a	Retrospective	0.011	0.000	−0.138	0.250	0.111
Expt 1b	Prospective	0.016	0.001	−0.290	0.227	−0.084
Expt 1c	Retrospective	0.004	0.000	n/a	−0.129	n/a
Expt 1c	Prospective	0.002	0.000	n/a	0.081	n/a
Expt 2	Retrospective	0.010	0.000	0.148	0.209	0.398
Expt 2	Prospective	0.027	0.002	−0.227	0.178	−0.052
Expt 3	Retrospective	0.001	0.000	0.049	−0.071	−0.024

The 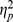 and 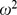
columns show the effect sizes for the effect of temporal structure on duration estimate in each experiment/condition. The effects for the prospective and retrospective judgment data from Experiments 1c and 2 have been analyzed separately, so each analysis is based on a one-way design and the partial eta-squared values shown here are identical to eta-squared. Note also that the calculation of omega-squared assumes a balanced design, but there were slightly unequal cell-sizes in Experiment 3. The last 3 columns show the standardized differences between means g* [64] calculated using the metafor package for R [46]. Acc = accelerating, Dec = Decelerating; Con = Constant-rate.

Our samples were large and our studies were highly-powered. Previous comparisons of accelerating, decelerating, and constant-rate stimuli found a median effect size of 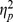 = .53 for 5 studies examining judgments of moving shapes [9] and 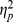 = .21 for 4 studies examining judgments of brief tone sequences [10] (the latter paper also reports additional experiments with similar effect sizes in its Supplementary materials). The power to detect such effects is more than 99.9% for every experiment reported here [47]. Even the smallest of the effects in the aforementioned papers was 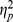 = .10, and the power to detect such an effect was 93.9%, 93.9%, 99.7%, 97.2%, and 93.7% for Experiments 1a-3, respectively. We also computed Bayes factors [48]–[51] for the duration estimates using a Zellner-Siow *g* prior [52] proposed as a default for ANOVA-type designs [53], [54]. These Bayes factors were computed using a modified version of the code provided by Wetzels and colleagues [53] and are shown in Table 5; in every case, the data favour the null hypothesis by at least 10 to 1.

**Table 5:** Bayes factors for the duration estimates of Experiments 1a-3.

Experiment	Bayes Factor
1a	68.2
1b	48.5
1c – Retrospective Judgments	10.3
1c – Prospective Judgments	11.7
2– Retrospective Judgments	28.9
2– Prospective Judgments	57.2
3	127.9

The Bayes factor is the probability of the observed data under the null hypothesis divided by the probability of the data under the distribution of alternative hypotheses specified by the Zellner-Siow *g* prior. Values greater than 1 indicate support for the null hypothesis that there is no effect of temporal structure on duration estimates. Values greater than 10 are often labelled “strong” evidence for the null; values greater than 30 are “very strong” evidence [48].

We must be cautious about generalizing these null results. Other stimuli and tasks might show a different pattern. Moreover, although the changes in tempo that we used were clearly noticeable, stronger manipulations might produce a significant effect. Our goal in reporting the current experiments is to provide a first investigation of how changes in the pace of external events influence one-off estimates of duration, and our results will be a useful contribution to subsequent meta-analyses of this issue.

If future work confirms our findings, then the null effect of changes in tempo will help constrain theoretical accounts of duration judgment. There are many accounts of temporal judgment, and it seems premature to engage in a lengthy discussion of all of them at this point. Rather, we briefly outline a few basic ideas. Considering first retrospective judgments, we noted above that many accounts have posited that remembered duration is positively related to the “storage size” [19], degree of segmentation [20], [21], or number of contextual changes [22] during the to-be-recalled interval (see also [1] for a change-based account of active timing). The null effects reported here suggest that, if these accounts are correct, then tempo changes do not add complexity or require more changes in processing than constant-rate event sequences. That is, the complexity/degree of segmentation/amount of change that determines a remembered duration seems to be defined in terms of first order change (the mean rate of events during the interval), not second order change (changes in the rate of events during the interval). Likewise, we found no indication that changes in tempo disrupt the coherence of the stimulus/event sequence in such a way as to impair duration estimates relative to constant-tempo stimuli.

Turning to prospective judgments, we noted in the Introduction that many theorists assume intentional timing to be based on counting or accumulating the output of a pacemaker whose rate may be linked to the rate of external stimulation. The null effects found here suggest either that the pacemaker rate is unaffected by external tempo, or that any coupling is linear (such that subjective duration depends only on the total number of external events in a given time period). Likewise, under the change-based account of Beckmann and Young [1], the current data argue that it is first-order change, not second-order change, which determines judged duration.

Our experiments found no significant differences between prospective and retrospective judgments. This stands in contrast to the more usual finding of longer and more accurate judgments in the prospective paradigm (e.g., [13], [55]). The reason for this difference is not clear. Possibly our forewarning was not a strong enough manipulation; instructions which more forcefully emphasized the need for an accurate temporal judgment in the prospective condition might have produced different results. In any case, our data will again make a useful contribution to meta-analyses of this effect [56].

Although we found no effect of temporal structure, studies using brief, meaningless stimuli which participants judge many times in a within-subject experiment show large effects (e.g., [6], [9]). Why the difference? One explanation is that psychophysical studies reduce noise, maximizing experimental effects (although it is perhaps unlikely that the current studies would not even find a consistent trend, especially given the large sample sizes). Low-noise, psychophysical studies help illuminate basic aspects of perception, but the results are much less relevant to one-off judgments of moderate durations of the kind that people typically make outside the laboratory. Furthermore, presenting many similar items for judgment within an experimental session provokes inter-item comparisons and perceptual contrast which can strongly influence judgment [15]–[17], [57]–[59]. An alternative (not contradictory) possibility is that timing mechanisms are different for durations of a few seconds and for intervals of a few minutes. In particular, longer durations and more complex, naturalistic stimuli permit a greater diversity of judgment strategies than brief, impoverished items. Exploring these disparate strategies will be a key direction for future research. Relatedly, it will be important to establish whether factors such as modality [32], [33], intensity [31], and repetition/familiarity [60]–[63], which exert a profound influence on psychophysical studies of perceived duration, have a noticeable impact on one-off judgments of naturalistic stimuli.

## References

[1] Beckmann JS, Young ME (2009) Stimulus dynamics and temporal discrimination: Implications for pacemakers. Journal of Experimental Psychology: Animal Behavior Processes 35: 525–537. 19839705 10.1037/a0015891

[2] Brown SW (1995) Time, change, and motion: The effects of stimulus movement on temporal perception. Perception & Psychophysics 57: 105–116. 7885802 10.3758/bf03211853

[3] Nakajima Y (1987) A model of empty duration perception. Perception 16: 485–520. 3444731 10.1068/p160485

[4] Thomas EAC, Brown I (1974) Time perception and the filled duration illusion. Perception & Psychophysics 16: 449–458.

[5] Makin ADJ, Poliakoff E, Dillon J, Perrin A, Mullet T, et al (2012) The interaction between duration, velocity, and repetitive auditory stimulation. Acta Psychologica 139: 524–531. 22370503 10.1016/j.actpsy.2012.01.013

[6] Buffardi L (1971) Factors affecting the filled-duration illusion in the auditory, tactual, and visual modalities. Perception & Psychophysics 10: 292–294.

[7] Adams RD (1977) Intervening stimulus effects on category judgments of duration. Perception & Psychophysics 21: 527–534.

[8] Israeli N (1930) Illusions in the perception of short time intervals. Archives of Psychology 19: 5–47.

[9] Matthews WJ (2011) How do changes in speed affect the perception of duration? Journal of Experimental Psychology: Human Perception and Performance 37: 1617–1627. 21517218 10.1037/a0022193

[10] Matthews WJ (2013) How does sequence structure affect the judgment of time? Exploring a weighted sum of segments model. Cognitive Psychology 66: 259–282. 23395774 10.1016/j.cogpsych.2013.01.001

[11] Tobin S, Bisson N, Grondin S (2010) An ecological approach to prospective and retrospective timing of long durations: A study involving gamers. PLoS ONE 5: e9271. 20174648 10.1371/journal.pone.0009271 PMC2822850

[12] Tobin S, Grondin S (2012) Time perception is enhanced by task duration knowledge: Evidence from experienced swimmers. Memory & Cognition 40: 1339–1135. 22806428 10.3758/s13421-012-0231-3

[13] Bisson N, Tobin S, Grondin S (2012) Prospective and retrospective time estimates of children: A comparison based on ecological tasks. PLoS ONE 7: e33049. 22412982 10.1371/journal.pone.0033049 PMC3295787

[14] Grondin S (2010) Timing and time perception: A review of recent behavioral and neuroscience findings and theoretical directions. Attention, Perception, and Psychophysics 72: 561–582. 10.3758/APP.72.3.561 20348562

[15] Matthews WJ (2011) Can we use verbal estimation to dissect the internal clock? Differentiating the effects of pacemaker rate, switch latencies, and judgment processes. Behavioural Processes 86: 68–74. 20933588 10.1016/j.beproc.2010.09.006

[16] Brown GDA, McCormack T, Smith M, Stewart N (2005) Identification and bisection of temporal durations and tone frequencies: Common models for temporal and nontemporal stimuli. Journal of Experimental Psychology: Human Perception and Performance 31: 919–938. 16262489 10.1037/0096-1523.31.5.919

[17] Wearden JH, Ferrara A (1995) Stimulus spacing effects in temporal bisection by humans. Quarterly Journal of Experimental Psychology 48B: 289–310. 8532899

[18] Zakay D, Tsal Y, Moses M, Shahar I (1994) The role of segmentation in prospective and retrospective time estimation processes. Memory & Cognition 22: 344–351. 8007836 10.3758/bf03200861

[19] Ornstein RE (1969) On the experience of time. Harmondsworth, Middlesex: Penguin.

[20] Poynter WD (1983) Duration judgment and the segmentation of experience. Memory & Cognition 11: 77–82. 6855562 10.3758/bf03197664

[21] Poynter WD (1989) Judging the duration of time intervals: A process of remembering segments of experience. In: Levin I, Zakay D, editors. Time and human cognition: A life-span perspective. Amsterdam: Elsevier. 305–321.

[22] Block RA, Reed MA (1978) Remembered duration: Evidence for a contextual-change hypothesis. Journal of Experimental Psychology: Human Learning and Memory 4: 656–665.

[23] Boltz MG (1998) Task predictability and remembered duration. Perception & Psychophysics 60: 768–784. 9682603 10.3758/bf03206062

[24] Boltz MG (1998) The processing of temporal and nontemporal information in the remembering of event durations and musical structure. Journal of Experimental Psychology: Human Perception and Performance 24: 1087–1104. 9706710 10.1037//0096-1523.24.4.1087

[25] Creelman CD (1962) Human discrimination of auditory durations. Journal of the Acoustical Society of America 34: 582–593.

[26] Gibbon J, Church RM, Meck WH (1984) Scalar timing in memory. In: Gibbon J, Allan L, editors. Annals of the New York Academy of Sciences, Volume 423: Timing and time perception. New York: New York Academy of Sciences. 10.1111/j.1749-6632.1984.tb23417.x 6588812

[27] Killeen PR, Fetterman JG (1988) A behavioral theory of timing. Psychological Review 95: 274–295. 3375401 10.1037/0033-295x.95.2.274

[28] Rammsayer T, Ulrich R (2001) Counting models of temporal discrimination. Psychonomic Bulletin & Review 8: 270–277. 11495114 10.3758/bf03196161

[29] Treisman M (1963) Temporal discrimination and the indifference interval: Implications for a model of the “internal clock”. Psychological Monographs 77: 1–31. 10.1037/h0093864 5877542

[30] Wearden JH (1992) Temporal generalization in humans. Journal of Experimental Psychology-Animal Behavior Processes 18: 134–144. 10.1037//0097-7403.23.4.502 9335137

[31] Matthews WJ, Stewart N, Wearden JH (2011) Stimulus intensity and the perception of duration. Journal of Experimental Psychology: Human Perception and Performance 37: 303–313. 20731508 10.1037/a0019961

[32] Wearden JH, Edwards H, Fakhri M, Percival A (1998) Why “Sounds are judged longer then lights”: Application of a model of the internal clock in humans. Quarterly Journal of Experimental Psychology 51B: 97–120. 10.1080/713932672 9621837

[33] Jones LA, Poliakoff E, Wells J (2009) Good vibrations: Human interval timing in the vibrotactile modality. Quarterly Journal of Experimental Psychology 62: 2171–2186. 10.1080/17470210902782200 19370453

[34] Penton-Voak IS, Edwards H, Percival A, Wearden JH (1996) Speeding up an internal clock in humans? Effects of click trains on subjective duration. Journal of Experimental Psychology: Animal Behavior Processes 22: 307–320. 8691161 10.1037//0097-7403.22.3.307

[35] Jones LA, Allely CS, Wearden JH (2011) Click trains and the rate of information processing: Does “speeding up” subjective time make other processes run faster? Quarterly Journal of Experimental Psychology 64: 363–380. 10.1080/17470218.2010.502580 20737353

[36] Kaneko S, Murakami I (2009) Perceived duration of visual motion increases with speed. Journal of Vision 9: 1–12. 10.1167/9.7.14 19761329

[37] Zakay D, Block RA (1997) Temporal cognition. Current Directions in Psychological Science 6: 12–16.

[38] Jones MR, Boltz M (1989) Dynamic attending and responses to time. Psychological Review 96: 459–491. 2756068 10.1037/0033-295x.96.3.459

[39] Brown SW (1997) Attentional resources in timing: Interference effects in concurrent temporal and nontemporal working memory tasks. Perception & Psychophysics 59: 1118–1140. 9360484 10.3758/bf03205526

[40] Zauberman G, Levav J, Diehl K, Bhargave R (2010) 1995 feels so close yet so far: The effect of event markers on subjective feelings of elapsed time. Psychological Science 21: 133–139. 20424034 10.1177/0956797609356420

[41] Droit-Volet S, Bigand E, Ramos D, Bueno JLO (2010) Time flies with music whatever its emotional valence. Acta Psychologica 135: 226–232. 20674884 10.1016/j.actpsy.2010.07.003

[42] Forster KI, Forster JC (2003) DMDX: A windows display program with millisecond accuracy. Behavior Research Methods, Instruments, & Computers 35. 10.3758/bf03195503 12723786

[43] Peirce JW (2007) PsychoPy - Psychophysics software in Python. Journal of Neuroscience Methods 162: 8–13. 17254636 10.1016/j.jneumeth.2006.11.017 PMC2018741

[44] Tabachnick BG, Fidell LS (2007) Using Multivariate Statistics. London: Pearson.

[45] R Core Team (2012) R: A language and environment for statistical computing. http://www.R-project.org.

[46] Viechtbauer W (2010) Conducting meta-analyses in R with the metafor package. Journal of Statistical Software 36: 1–48.

[47] Faul F, Erdfelder E, Lang A-G, Buchner A (2007) G*Power 3: A flexible statistical power analysis program for the social, behavioral, and biomedical sciences. Behavior Research Methods 39: 175–191. 17695343 10.3758/bf03193146

[48] Wagenmakers E-J, Wetzels R, Borsboom D, van der Maas HLJ (2011) Why psychologists must change the way they analyze their data: The case of Psi: Comment on Bem (2011). Journal of Personality and Social Psychology 100: 426–432. 21280965 10.1037/a0022790

[49] Kass RE, Raftery AE (1995) Bayes factors. Journal of the American Statistical Association 90: 773–795.

[50] Rouder JN, Speckman PL, Sun D, Morey RD, Iverson G (2009) Bayesian *t* tests for accepting and rejecting the null hypothesis. Psychonomic Bulletin & Review 16: 225–237. 19293088 10.3758/PBR.16.2.225

[51] Matthews WJ (2011) What might judgment and decision making research be like if we took a Bayesian approach to hypothesis testing? Judgment and Decision Making 6: 843–856.

[52] Zellner A, Siow A (1980) Posterior odds ratios for selected regression hypotheses. In: Bernardo JM, DeGroot MH, Lindley DV, Smith AFM, editors. Bayesian statistics: Proceedings of the first international meeting held in Valencia. Valencia, Spain: University of Valencia Press. 585–603.

[53] Wetzels R, Grasman RPPP, Wagenmakers E-J (2012) A default Bayesian hypothesis test for ANOVA designs. The American Statistician 66: 104–111.

[54] Liang F, Paulo R, Molina G, Clyde MA, Berger JO (2008) Mixtures of *g* priors for Bayesian variable selection. Journal of the American Statistical Association 103: 410–423.

[55] Brown SW, Stubbs DA (1992) Attention and interference in prospective and retrospective timing. Perception 21: 545–557. 1437469 10.1068/p210545

[56] Block RA, Zakay D (1997) Prospective and retrospective duration judgments: A meta-analytic review. Psychonomic Bulletin & Review 4: 184–197. 21331825 10.3758/BF03209393

[57] Matthews WJ, Stewart N (2009) The effect of interstimulus interval on sequential effects in absolute identification. Quarterly Journal of Experimental Psychology 62: 2014–2029. 10.1080/17470210802649285 19235096

[58] Matthews WJ, Stewart N (2009) Psychophysics and the judgment of price: Judging complex objects on a non-physical dimension elicits sequential effects like those in perceptual tasks. Judgment and Decision Making 4: 64–81.

[59] Ward LM, Lockhead GR (1970) Sequential effects and memory in category judgments. Journal of Experimental Psychology 84: 27–34.

[60] Pariyadath V, Eagleman DM (2007) The effect of predictability on subjective duration. PLoS ONE 2: e1264. 18043760 10.1371/journal.pone.0001264 PMC2082074

[61] Pariyadath V, Eagleman DM (2012) Subjective duration distortions mirror neural repetition suppression. PLoS ONE 7: e49362. 23251340 10.1371/journal.pone.0049362 PMC3521010

[62] Matthews WJ (2011) Stimulus repetition and the perception of time: The effects of prior exposure on temporal discrimination, judgment, and production. PLoS ONE 6: e19815. 21573020 10.1371/journal.pone.0019815 PMC3090413

[63] Tse PU, Intriligator J, Rivest J, Cavanagh P (2004) Attention and the subjective expansion of time. Perception & Psychophysics 66: 1171–1189. 15751474 10.3758/bf03196844

[64] Hedges LV, Olkin I (1985) Statistical methods for meta-analysis. Orlando: Academic Press.

